# Colorectal cancer‐derived FGF19 is a metabolically active serum biomarker that exerts enteroendocrine effects on mouse liver

**DOI:** 10.1002/1878-0261.70212

**Published:** 2026-02-03

**Authors:** Jordan M. Beardsley, Michael W. Rohr, Joseph A. Goode, Sai Preethi Nakkina, Jignesh G. Parikh, Deborah A. Altomare

**Affiliations:** ^1^ Burnett School of Biomedical Sciences, College of Medicine University of Central Florida Orlando FL USA; ^2^ Department of Pathology Orlando VA Medical Center FL USA; ^3^ Present address: Department of Pharmacology, College of Medicine University of Texas Southwestern Dallas TX USA; ^4^ Present address: Department of Pathology and Laboratory Medicine, Perelman School of Medicine University of Pennsylvania Philadelphia PA USA

**Keywords:** colon cancer, enterohepatic signaling, fibroblast growth factor‐19, meta‐analysis, serum marker

## Abstract

Despite having excellent prognosis when detected early, colorectal cancer (CRC) remains a leading cause of cancer‐related deaths globally. Screening remains an important contributor to CRC survival, but the cost and invasiveness of traditional imaging methodologies can hinder patient compliance. A blood‐based approach would be more convenient and accessible, but reliable serum markers are lacking. In this study, the peptide enteroendocrine hormone Fibroblast Growth Factor 19 (FGF19) was identified as an attractive marker for CRC through a meta‐transcriptomic analysis. While its pro‐tumor effects are documented, FGF19's utility as a blood serum marker for CRC is not well defined. Studies presented here show that FGF19 is constitutively expressed and secreted in 3 of 5 CRC cell lines, and secreted levels increase with seeding density. A subcutaneous CRC cell line‐derived xenograft model revealed that FGF19 is detectable in serum of mice injected with FGF19‐positive, but not negative, CRC cells at levels corresponding to tumor volume. Enteroendocrine effects of tumor‐derived FGF19, including suppression of bile acid synthesis, are evident in liver samples via RNA sequencing and validated by RT‐PCR. Notably, the hepatic response to CRC‐secreted FGF19 has not been explored prior to this study even though FGF19 is a key regulator of hepatic cholesterol metabolism and bile acid homeostasis. Collectively, these findings support the clinical utility of FGF19 as a putative serum marker for CRC and provide important evidence that CRC‐derived FGF19 can modulate liver physiology consistent with the enteroendocrine function of FGF19.

## Graphical abstract

Meta‐transcriptome analysis identified FGF19 as a peptide enteroendocrine hormone associated with colorectal cancer prognosis. *In vivo* xenograft models showed release of FGF19 into the blood at levels that correlated with tumor volumes. Tumoral‐FGF19 altered murine liver metabolism through FGFR4, thereby reducing bile acid synthesis and increasing glycogen production. Collectively, FGF19 may serve as an accessible blood‐based marker for colorectal cancer.

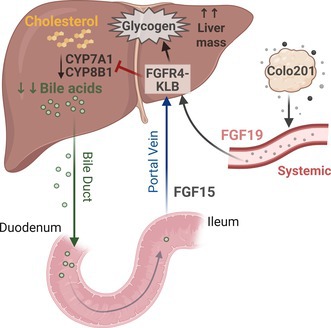

**Abbreviations**
AUC: area under the curve
BAs: bile acids
BSA: bovine serum albumin
CA: cholic acid
CDCA: chenodeoxycholic acid
CDX: cell‐derived xenograft
CRC: colorectal cancer
CYP7A1: cytochrome P450 family 7 subfamily A member 1
CYP8B1: cytochrome P450 family 8 subfamily B member 1
DCA: deoxycholic acid
DE: differential expression
DEG: differentially expressed gene
DFS: disease‐free survival
ELISA: enzyme‐linked immunosorbent assay
EMT: epithelial to mesenchymal transition
FBS: fetal bovine serum
FDR: false discovery rate
FGF15: fibroblast growth factor 15
FGF19: fibroblast growth factor 19
FGFR4: fibroblast growth factor receptor 4
FXR: farnesoid‐x‐receptor
GAPDH: glyceraldehyde‐3‐phosphate dehydrogenase
GO: gene ontology
GTEx: genome tissue expression dataset
H&E: hematoxylin and eosin
HCC: hepatocellular carcinoma
IHC: immunohistochemistry
INSIG1: insulin induced gene 1
IPA: ingenuity pathway analysis
KEGG: kyoto encyclopedia of genes and genomes
KLB: klotho beta
LCA: lithocholic acid
LXR: liver‐x‐receptor
MCA: muricholic acid
MKI67: marker of proliferation KI67
NRF2: nuclear factor‐erythroid factor 2‐related factor 2
NSG: NOD scid gamma
OS: overall survival
PAS: Periodic Acid‐Schiff
PBS: phosphate‐buffered saline
PCA: principal component analysis
pEH: peptide enteroendocrine hormone
PXR: pregnane‐x‐receptor
qRT‐PCR: quantitative real‐time‐polymerase chain reaction
RIN: RNA integrity number
RNAseq: RNA sequencing
ROC: receiver operator characteristic
RXR: retinoid‐x‐receptor
SC: subcutaneous
SCAP: sterol regulatory element‐binding protein cleavage‐activating protein
SREPB2: sterol regulatory element‐binding protein 2
TCGA‐COADREAD: The Cancer Genome Atlas‐ Colorectal adenocarcinoma
TME: tumor microenvironment

## 1 Introduction

Intensified screening efforts have significantly reduced colorectal cancer (CRC) mortality largely due to a shift in using colonoscopy as the go‐to screening method for boosting compliance. Unfortunately, one‐third of eligible Americans still do not follow recommended screening guidelines, a statistic that is expected to worsen with the inclusion of the less compliant younger 45–50 age demographic [1]. A survey of 172 patients found that of those refusing colonoscopy, 97% preferred non‐invasive alternatives, with the majority (83%) favoring a blood‐based test [2]. However, reliable blood‐based biomarkers for colorectal cancer are currently lacking. Using peptide enteroendocrine hormones (pEHs), which are proteins secreted by enteroendocrine cells that are readily detected in blood, aberrant tumor‐derived pEH detection could be used to facilitate the diagnosis of CRC.

We identified Fibroblast Growth Factor 19 (FGF19) as a top pEH candidate. FGF19 has a normal non‐canonical endocrine FGF function to primarily regulate hepatic *de novo* synthesis of bile acids (BAs), amongst other metabolic functions [3]. Following a meal, 95–98% of BAs expelled into the duodenum are reabsorbed at the ileum and transported back to the liver via portal circulation. Within ileocytes, BAs interact with Farnesoid‐X‐Receptor (FXR), in turn stimulating FGF19 transcription and secretion [4]. FGF19 is delivered to hepatocytes and interacts with surface Fibroblast Growth Factor Receptor 4 (FGFR4) and Klotho Beta (KLB) coreceptor, resulting in endocrine regulation of lipid and energy homeostasis and glycogen, protein, and importantly, BA synthesis. Synthesis of BAs from cholesterol occurs predominantly through CYP7A1 and CYP8B1, which are the chief enzymatic targets of FGF19‐activated FGFR4.

A role for FGF19 in promoting tumorigenesis is also emerging. Tumor FGF19 overexpression was first described for hepatocellular carcinoma (HCC) where investigations show that FGF19 drives tumor progression via dysregulation of FGFR4 signaling [5, 6, 7, 8, 9, 10, 11]. Aside from HCC, FGF19 also contributes to the aggressiveness of cancer types such as breast, lung, head and neck and has additionally been detected in the serum of prostate, acute myeloid leukemia, thyroid, nasopharyngeal, and CRC patients [12, 13, 14, 15, 16, 17, 18, 19]. Specific pro‐tumor functions of FGF19 include promotion of epithelial to mesenchymal transition (EMT), chemoresistance, and lesion advancement which are disrupted upon neutralization of FGF19 in HCC and CRC models [20, 21, 22, 23, 24, 25, 26, 27, 28, 29]. There is a gap in knowledge regarding CRC secretory patterns of FGF19 in blood serum concurrently with how the liver responds to persistent ectopic stimulation from this aberrant tumor‐derived growth factor.

Collectively, this study used *in silico* analyses, as well as *in vitro* and *in vivo* testing of CRC tumor models to assess FGF19's potential applicability as a serum marker. *In vivo* testing led to the evaluation of serum FGF19 levels over time and relative to tumor size. Importantly, the *in vivo* testing also revealed that tumor‐derived FGF19 deregulated normal metabolic BA signaling pathways and potentially glycogen storage in the liver. These novel findings highlight evidence for tumor‐derived FGF19's secretion from the tumor environment, detection of its presence in blood, and its systemic effects to dysregulate normal metabolic pathways in hepatic tissue.

## 2 Material and methods

### 2.1 Meta‐transcriptomics

For normal colon epithelial and CRC samples, RNAseq data were obtained from the Genotype Tissue Expression (GTEx) consortium and TCGA‐COADREAD, respectively [30, 31]. For adenoma samples, transcriptomic profiles were obtained from our openly accessible and published Meta‐dataset [32]. FGF19 biomarker strength was determined by performing receiver operator characteristic (ROC) analysis on *FGF19* expression profiles between normal and CRC samples from the TCGA‐COADREAD cohort. Maximal sensitivity and specificity values were calculated using the Youden Index. Differences in *FGF19* expression between indicators of poor prognosis such as pathologic stage, tumor stage, metastasis, disease progression, and treatment failure were also assessed from the TCGA‐COADREAD cohort. The impact of *FGF19* on patient survival was determined via Kaplan–Meier analysis of overall survival (OS) and disease‐free survival (DFS) for low‐ and high‐*FGF19* expression groups. ESTIMATE scores of immune and stromal signatures (R package 1.0.13) were determined for TCGA‐COAD and E‐MTAB‐10089 samples [33]. *FGF19* expression was then examined by Spearman correlation with the ESTIMATE, immune, and stromal scores.

### 2.2 Cell lines

Cell lines SW620 (RRID:CVCL_0547), HT‐29 (RRID:CVCL_0320), and Colo201 (RRID:CVCL_1987) were purchased from American Type Culture Collection (ATCC, Manassas, VA, USA). Caco‐2 (RRID:CVCL_0025), HIEC‐6 (RRID:CVCL_6C21), and HCT116 (RRID:CVCL_0291) were gifted by Dr. Justine Tigno‐Aranjuez at the University of Central Florida in 2019. Colo201 was grown in RPMI and HCT116 was grown in DMEM with 1% Glutamine. Media was supplemented with 1% penicillin/streptomycin and 10% fetal bovine serum (FBS). Authentication was performed by ATCC via short tandem repeat analysis within 3 years of the time of use. Negative mycoplasma results were confirmed by PCR (Southern Biotech, Birmingham, AL, USA) using JumpStart DNA polymerase (Sigma Aldrich, St. Louis, MO, USA). Cell lines were grown in a humidified 37 °C incubator with 5% CO_2_ and used for experiments under 20 passages.

### 2.3 Quantitative RT‐PCR

RNA was extracted from cell lines or ~50 mg of xenograft, liver, and ileal tissue using TRIzol reagent (Invitrogen ThermoFisher Scientific, Waltham, MA, USA) following the manufacturer's instructions. RNA concentration and purity were determined using a Nanodrop 2000 spectrophotometer (ThermoFisher). Approximately 1 μg RNA was reverse transcribed using the Maxima First Strand cDNA kit (ThermoFisher). Samples were prepared for quantitative PCR using the Fast SYBR Green reagent (Applied Biosystems ThermoFisher) and amplified in a QuantStudio7 instrument (Applied Biosystems). All genes were normalized either to human GAPDH (for human cell line and xenograft samples) or mouse Gapdh (for organ samples), and relative expression was determined using the $2^{-\Delta \Delta C_{T}}$ method. Primer sequences are listed below

**Table:** 

Quantitative RT‐PCR Primers		
	Forward primer (5′ → 3′)	Reverse primer (5′ → 3′)
**Human genes**		
*FGF19*	GTCCCAGCTTTGAGAAGTAA	GACTCAGGACTGTTCTTGTAG
*GAPDH*	GTATGACAACAGCCTCAAGAT	GTCCTTCCACGATACCAAAG
**Mouse genes**		
*Fgf15*	CAGTCTGTGTCAGATGAAG	GGAAGCAGTTGGAGACATAG
*Gapdh*	CTGAGTATGTCGTGGAGTCTAC	GTTGGTGGTGCAGGATGCATTG
*Cyp7a1*	CACAAACTCCCTGTCATACC	CACTTGGGTCTATGCTTCTG
*Cyp8b1*	CTGAGGGAGCAAGGAATAG	GGAATAAGAGGACCCAGAAA
*Ki67*	CAGCAGCAGATGAGTGATAC	TGATGGGCTCAGGTATGT
*Afp*	CCAGCTATCTGTGTTTCTGG	TGTCTTTCCACTCCACTTTG
*Gpc3*	GGAACGGGATGAAGAATCAG	CTCAGGAGCTGGTTAATGTG

### 2.4 Western blot analysis

Cell lysates were prepared in RIPA buffer (Santa Cruz Biotechnology or SCBT, Santa Cruz, CA, USA) and total protein was quantified via Pierce BCA assay or Pierce Bradford assay (ThermoFisher). Thirty μg of protein was separated by SDS/PAGE and transferred to a 0.45 μm nitrocellulose membrane (Bio‐Rad Laboratories, Hercules, CA, USA). Blots were blocked with 5% BSA (Sigma Aldrich) for 1 h and incubated with primary antibodies against Phospho‐FGFR4 (Tyr642) (Abcam, Cambridge, UK, ab192589), FGFR4 (SCBT, #sc‐136988), FGF19 (SCBT, #sc‐390621), Phospho‐GSK‐3α/β (Ser21/9) (#9331) (Cell Signaling Technology or CST, Danvers, MA, USA), GSK‐3α/β (CST, #5676), β‐tubulin (CST, #2128), and β‐actin (SCBT, #sc‐8432) overnight at 4 °C. Blots were then incubated with DyLight 488 anti‐mouse secondary antibodies (ThermoFisher, #35502) or HRP‐conjugated anti‐rabbit IgG (Jackson ImmunoResearch, #111‐035‐003) or HRP‐conjugated anti‐rabbit IgG (Jackson ImmunoResearch, #111‐035‐003) for 1 h at room temperature, and visualized with Pierce ECL western blotting substrate (ThermoFisher, #32209) for imaging using a ChemiDoc instrument (Bio‐Rad). Primary and secondary antibodies were used at 1 : 1000 and 1 : 10 000 dilutions, respectively. Quantification by densitometry of western blot bands was performed using ImageJ [34].

### 2.5 ELISA

For measuring secretory FGF19 levels, 1 × 10^6^ cells were seeded on a 6‐well plate and starved overnight in serum‐free medium. The following day, cells were incubated with fresh serum‐free medium for an additional 48 h. Conditioned media was then collected, centrifuged at 15 000 ***g*** for 10 min at 4 °C to remove cellular debris, and stored at −80 °C until use. Sandwich ELISA was performed on aliquots of the conditioned media diluted in PBS using the Human FGF19 ELISA Kit EZ‐Set (Boster Bio, Pleasanton, CA, USA) following the manufacturer's instructions. For measuring FGF19 levels in murine blood, snap‐frozen serum samples from each collection time point were thawed, diluted in PBS, and subjected to ELISA.

### 2.6 Animal studies

All experiments using mice were approved by the University of Central Florida Institutional Animal Care and Use Committee (IACUC) and performed in accordance with the National Institutes of Health (NIH) Guide for the Care and Use of Laboratory Animals. Animals were housed in a specific pathogen‐free facility, in rooms with a 12‐h light–dark cycle. Animals were housed at a maximum of 5 same‐sex adult mice per individual ventilated cage (Green line, Tecniplast, Milan, IT). 1 × 10^6^ mycoplasma‐free HCT116 and Colo201 cells were suspended in phosphate‐buffered saline (PBS) and injected subcutaneously into the right flanks of immunodeficient NOD.Cg‐*Prkdc* ^*scid*^ *Il2rg* ^*tm1Wjl*^/SzJ (NSG; RRID:IMSR_JAX:005557) mice, sourced from Jackson Laboratory (Bar Harbor, ME, USA). Two independent studies were performed, including a pilot study of 8 total mice (*n* = 4 per group) (IACUC protocol number: PROTO202000182) and a second study of 40 total mice (*n* = 20 per group) (IACUC protocol number: IPROTO202300001), both using equal numbers of male and female 6‐ to 8‐week‐old age‐matched mice that were randomized by cage for receiving one of the tumor cell lines. Cell‐derived xenograft (CDX) tumor dimensions were measured with calipers weekly for 5 weeks and were used to compute tumor volume using the formula (volume = length × width^2^/2). Overnight fasted blood samples (~100 μL up to weekly per mouse) were collected into serum separator tubes (Microtainer, BD Biociences, Franklin Lakes, NJ, USA). Serum was isolated by centrifugation at 10 000 ***g*** for 10 min. At study endpoint, equal numbers of overnight fasted and *ad libitum* fed mice were anesthetized with isoflurane and underwent cardiac puncture for blood collection. Immediately following secondary euthanasia, CDX tumors and livers were collected for RNA and histology. Distal small intestines were collected for RNA from a subset of *ad libitum* fed animals.

### 2.7 Histological analysis

Collected CDX tumors and livers were fixed for at least 24 h in 10% neutral buffered formalin (Surgipath Medical Industries, Northbrook, IL, USA). Samples were processed and embedded in paraffin. Multiple 5‐micron thick sections were isolated using a rotary microtome and baked onto glass slides for 1 h at 60 °C. Tissue sections were stained with hematoxylin and eosin (H&E) (Surgipath). Liver sections also were stained with a Periodic Acid‐Schiff (PAS) kit (StatLab, McKinney, TX, USA) for detection of glycogen, per manufacturer's instructions. Automated immunohistochemical staining was performed using the Quantum HDx Immunostainer (StatLab) with antibodies recognizing FGF19 (SCBT, #sc‐390621) or Ki67 (Abcam, #ab16667), using epitope retrievals of sodium citrate pH 6 or EDTA pH 8, respectively. Stained slides were assessed by a pathologist and imaged at 10×, 20×, and/or 40× magnifications using a BZ‐X800 microscope (Keyence, Osaka, Japan). PAS staining was quantified using ImageJ with the plug‐in Color Deconvolution 2 and measured for area fraction percentage [34, 35, 36, 37].

### 2.8 Bile acid quantification

Bile acids were quantitated from serum samples at 1 : 15 dilution in deionized water using the Total Bile Acid Assay Kit (Cell Biolabs, San Diego, CA, USA) following the manufacturer's instructions.

### 2.9 RNA sequencing and data processing

Sequencing libraries were prepared from murine liver mRNA samples following an Illumina (San Diego, CA, USA) TruSeq‐stranded‐mRNA protocol. RNA integrity was assessed using an Agilent Technologies 2100 Bioanalyzer (Santa Clara, CA, USA). Samples with RIN > 7 were subjected to 150 bp paired‐end sequencing on an Illumina NovaSeq 6000 system by LC Biosciences (Houston, TX, USA). Data processing including filtering, read mapping and alignment, and transcript assembly was performed using LC Biosciences custom informatics pipeline (Fig. S1).

### 2.10 Batch correction

Batch correction was performed on the original count matrix using ComBat‐seq (part of the sva R package) with covariates designated as CDX group and batches set as mouse sex. All other parameters were set to default [38]. Batch effect normalization was validated by PCA, which was performed on the top 10% variable genes using the edgeR package [39].

### 2.11 Differential expression analysis

Differential expression (DE) analysis was performed using edgeR. The counts matrix was first normalized by library size and filtered for low variant transcripts. Common, trended, and tagwise dispersions were then calculated using the integrated Cox‐Reid profile‐adjusted likelihood method and fitted to a generalized linear model. A quasi‐likelihood *F*‐test was performed to estimate gene log fold change (logFC) and *P*‐values. False discovery rates (FDR) were calculated using the Benjamini–Hochberg method. DE genes (DEGs) were considered when FDR < 1%. Volcano plots were used to visualize the distribution of DEGs for both the original and batch‐corrected datasets. A heatmap of scaled expression values of the top 20 up‐ and downregulated DEGs was generated using the pheatmap R package. Samples and genes were hierarchically clustered based on Euclidean‐distance weighting and Pearson correlation.

### 2.12 Functional enrichment analysis

Functional enrichment of gene ontology (GO) and Kyoto Encyclopedia of Genes and Genomes (KEGG) terms was performed using the integrated goana and kegga functions, respectively. Dot plots were used to visualize select significant terms and Rich Factor, which is defined as the number of DEGs divided by the total number of term‐associated genes.

Ingenuity Pathway Analysis (IPA) was used to further analyze changes in activity, mechanism, and relationships of major transcriptional events. IPA predicts associations within the data by comparing results to literature as well as a curated Knowledge Base. The list of DEGs and corresponding logFC, *P*‐values, and FDRs were submitted to IPA for conventional core expression analysis. A table of canonical pathways with a −log(FDR) > 2.0 as well as their interconnectivity was used for visualization. Likewise, the top regulator network was used to visualize the most impacted downstream pathways and associated upstream regulators.

### 2.13 FGF19 signaling signature analysis

Pre‐processed RNAseq data were obtained from GSE158359, and DE analysis was performed using edgeR [40]. Upregulated DEGs were designated as the FGF19‐induced signature while downregulated DEGs were considered the FGF19‐repressed signature. To calculate the signature score, median‐centered expression values in our RNAseq dataset were averaged across all signature genes for all samples individually, as done previously [41].

### 2.14 Statistics

All analyses were performed either in R or GraphPad Prism. For meta‐transcriptomics, significance was computed using Welch's *t*‐test for two groups or one‐way ANOVA corrected with post hoc Tukey's test for more than two groups. For survival analysis, log‐rank and Cox proportional hazard tests were used to compute significance and hazard ratios, respectively. Biomarker strength was computed as area under the curve (AUC) via ROC analysis. For *in vitro* studies, results are representative of at least three biological replicates and reported as mean ± SD. *In vivo* results were obtained from *n* = 20–24 per group, unless otherwise specified, and associated RNAseq results use *n* = 4 mice per group, which are reported as mean ± SEM. Tumor volume measurements and serum FGF19 levels were compared between groups across different time points using a mixed effects model two‐way ANOVA followed by Šídák multiple comparisons testing. Spearman coefficient was used for correlation analyses. Significance for all other grouped *in vivo* comparisons was determined via unpaired Student's *t*‐test. *P* < 0.05 is considered significant, where *P < 0.05, **P < 0.01, ***P < 0.001, and ****P < 0.0001.

## 3 Results

### 3.1 FGF19 is a prognostic marker that is overexpressed in colorectal neoplasms

**Fig. 1:**
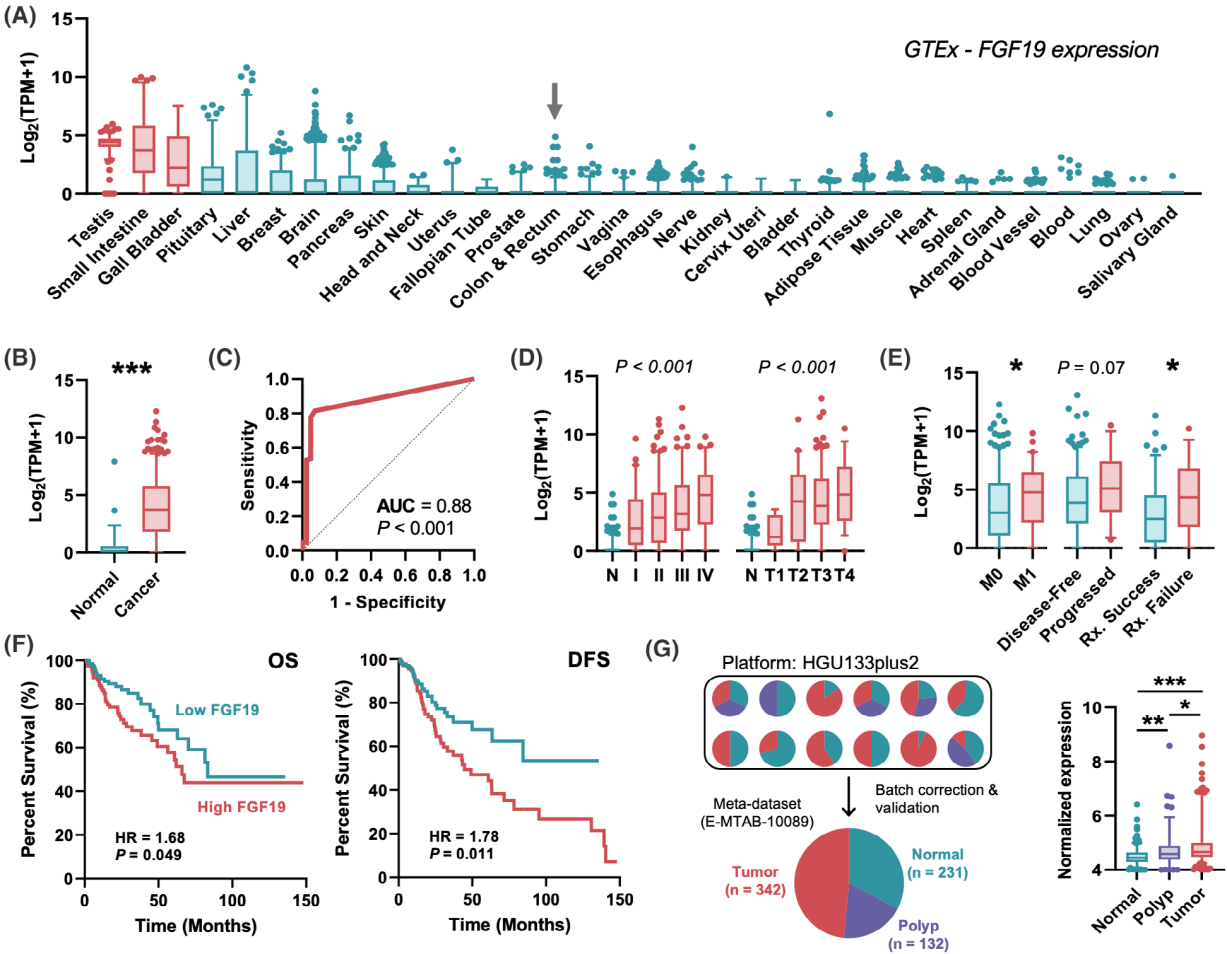
Meta‐transcriptomic analysis reveals FGF19 as a putative CRC biomarker. (A) Box plots illustrate the distribution of physiologic *FGF19* expression which is high in normal testis, small intestines, and gallbladder (red plots). *FGF19* is comparably low or not natively expressed in colon tissue (black arrow). (B) Box plots show that *FGF19* is overexpressed in CRC tissue but not in normal colon. Significance was calculated using Welch's *t*‐test. (C) ROC analysis of *FGF19* expression in CRC tissue shows *FGF19*'s strength as a putative biomarker. (D) Box plots of *FGF19* expression across pathologic (left) and tumor stages (right). Data from TCGA‐COADREAD cohort. Significance was calculated using a one‐way ANOVA. (E) Box plots of *FGF19* expression compared across different clinicopathologic variables. Significance was computed using Welch's *t*‐test. (F) Kaplan–Meier curves compare differences in overall (OS) (left) and disease‐free survival (DFS) (right) amongst patients with low‐ and high‐*FGF19*‐expressing tumors. Significance and hazard ratios were calculated via log‐rank and Cox proportional hazard tests, respectively. (G, left) Schematic detailing how the meta‐dataset, which contains normal, polyp, and CRC samples were generated. (G, right) Box plots show distribution of FGF19 expression in meta‐dataset samples. Significance was calculated using one‐way ANOVA with *post hoc* Tukey's test. All data were either from GTEx, TCGA‐COADREAD, or meta‐dataset cohorts. 5th to 95th percentile is shown for all box and whisker plots. Statistics: *P < 0.05, **P < 0.01, and ***P < 0.001.

First, we assessed whether normal physiological *FGF19* expression was tissue restricted. Using RNAseq data from the GTEx consortium, *FGF19* expression was mapped across tissue types. In contrast to low or undetectable expression in normal colon (Fig. 1A), *FGF19* expression was highly expressed in the tissues associated with BA recycling such as small intestines and gallbladder (red). *FGF19* was also found to be highly expressed in testicular tissue, but a function has yet to be described.

To evaluate FGF19's utility as a CRC biomarker, we queried expression profiles in normal colon, adenoma, and cancer tissue samples across publicly available RNA sequencing and microarray datasets. Analysis of RNAseq data from the TCGA‐COADREAD cohort showed that *FGF19* was low or absent in healthy tissue (*n* = 51) but significantly elevated in ~85% of CRC samples (*n* = 381, *P* < 0.0001), clearly demonstrating a CRC‐associated expression pattern (Fig. 1B). ROC analysis further showed *FGF19* as a strong predictive marker for CRC (AUC = 0.88, *P* < 0.0001) with a maximal sensitivity of 0.85 and specificity of 0.95 (Fig. 1C). Additional analysis revealed that *FGF19* expression increases stepwise with pathologic and tumor stages (Fig. 1D), while being associated with disease metastasis (*P* = 0.043), treatment failure (*P* = 0.028), and tended to be associated with progression (*P* = 0.071) (Fig. 1E). Furthermore, *FGF19* overexpression was negatively prognostic for patient overall survival (OS) and disease‐free survival (DFS) (Fig. 1F).

*FGF19* expression patterns also were assessed in adenoma tissue. Although the TCGA‐COADREAD cohort lacks such samples, our group recently compiled and validated a meta‐dataset containing normal (*n* = 231), adenoma (*n* = 132), and CRC (*n* = 342) samples by pooling transcriptome data across 12 independent microarray studies [32]. A schematic for the data collection is depicted (Fig. 1G, left). Analysis of the meta‐dataset revealed elevated *FGF19* expression in adenoma samples relative to normal tissue (*P* = 0.003), and *FGF19* expression levels were further increased in CRC tumors compared to adenoma samples (*P* = 0.022) (Fig. 1G, right). The ESTIMATE algorithm enables predictions of immune and stromal cells that make up the tumor microenvironment (TME) based upon gene expression data [33]. Within the TCGA‐COAD cohort (*n* = 524), *FGF19* expression was inversely correlated with ESTIMATE (*r* = −0.2343, *P*‐value < 0.0001), immune (*r* = −0.2337, *P*‐value < 0.0001), and stromal (*r* = −0.2062, *P*‐value < 0.0001) scores (Fig. S2A). Our meta‐dataset (*n* = 705) also had inverse correlation of *FGF19* expression with ESTIMATE (*r* = −0.1278, *P*‐value = 0.0007) and immune (*r* = −0.1773, *P*‐value < 0.0001) scores, although less strong (Fig. S2B). The subset of carcinoma samples (*n* = 342) from E‐MTAB‐10089 had weak inverse correlation with the immune score (*r* = −0.1123, *P*‐value = 0.0379) but not stromal or ESTIMATE scores (Fig. S2C).

### 3.2 FGF19 is secreted by CRC cells in a density‐dependent manner

**Fig. 2:**
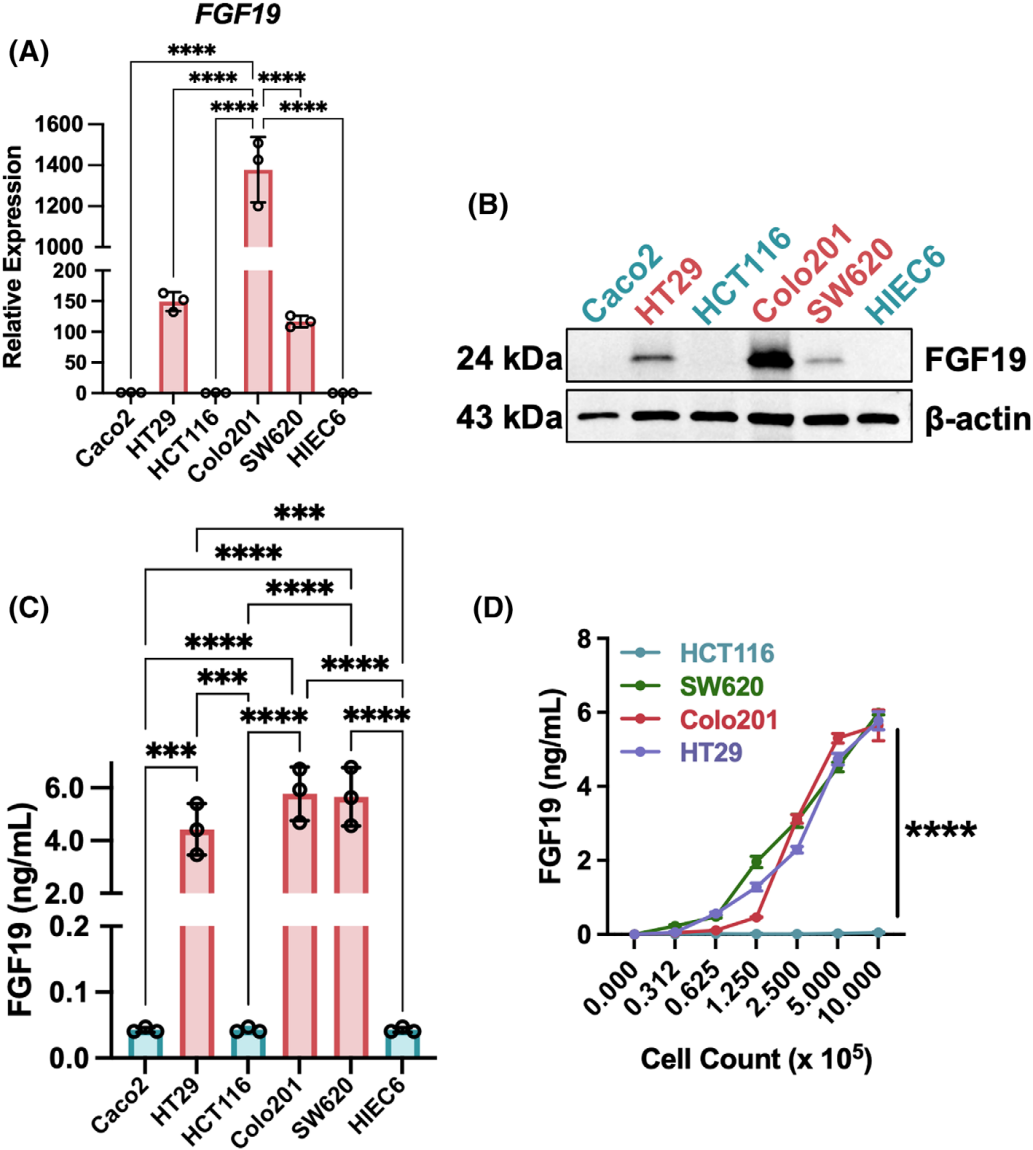
Ectopic FGF19 expression and secretion in CRC cells is linked *in vitro*. (A) Quantitative RT‐PCR analysis of *FGF19* expression across five CRC and one normal colon (HIEC6) cell line. Expression was normalized to *GAPDH* and set relative to Caco2 cells. Significance was determined by an ordinary one‐way ANOVA with Tukey's multiple comparisons test. (B) Western blot analysis of CRC cell lysates for FGF19 protein expression. (C) Basal FGF19 secretion across CRC cell lines as determined by ELISA of conditioned media. Significance was determined by an ordinary one‐way ANOVA with Tukey's multiple comparisons test. (D) Media FGF19 levels coincide with seeding density. Experiments were conducted under serum‐free conditions to prevent latent stimulation by bile acids contained within fetal bovine serum. SW620, HT29, and Colo201 were all significantly increased compared to HCT116 (*P* < 0.0001) starting at seeding density of 1.25 × 10^5^ through 10 × 10^5^. Significance was determined by an ordinary two‐way ANOVA with Dunnett's multiple comparisons test. All experiments were performed in triplicates and results are represented as mean ± SD. Statistics: ***P < 0.001 and ****P < 0.0001.

Based on FGF19's endocrine properties and ability to induce tumorigenesis via autocrine‐paracrine signaling with FGFR4, we hypothesized that secretion and expression were related [12, 13]. To test this, *FGF19* expression patterns were analyzed in a panel of CRC cell lines previously shown to express *FGF19* via qRT‐PCR analysis [21]. Colo201 cells had the highest *FGF19* expression, while HT29 and SW620 cells had detectable expression (Fig. 2A). In contrast, HCT116 and Caco2 cells did not have detectable *FGF19* expression. Western blot analysis of cell lysates yielded similar results (Fig. 2B). In both scenarios, FGF19 was not detected in the normal intestinal cell line HIEC6, as expected.

Next, we tested if FGF19 was actively secreted by CRC cells. Media obtained from cells incubated under serum‐free conditions was evaluated for FGF19 via sandwich ELISA. FGF19 was detected at ng·mL^−1^ concentrations only in media from FGF19‐expressing cell lines (Fig. 2C). We next tested whether secretion was correlated with cellular density since the detected levels of FGF19 by ELISA varied from differences originally observed in the cell lines by qRT‐PCR or western. FGF19 was quantified in media conditioned from cells at different seeding densities ranging from 3 × 10^4^ to 1 × 10^6^ cells. It was found that FGF19 levels in media increased with seeding density, and that FGF19‐positive cell lines had similar peak levels depending on cell number (Fig. 2D).

### 3.3 Cell‐derived colorectal tumors actively secrete FGF19 into serum *in vivo*

**Fig. 3:**
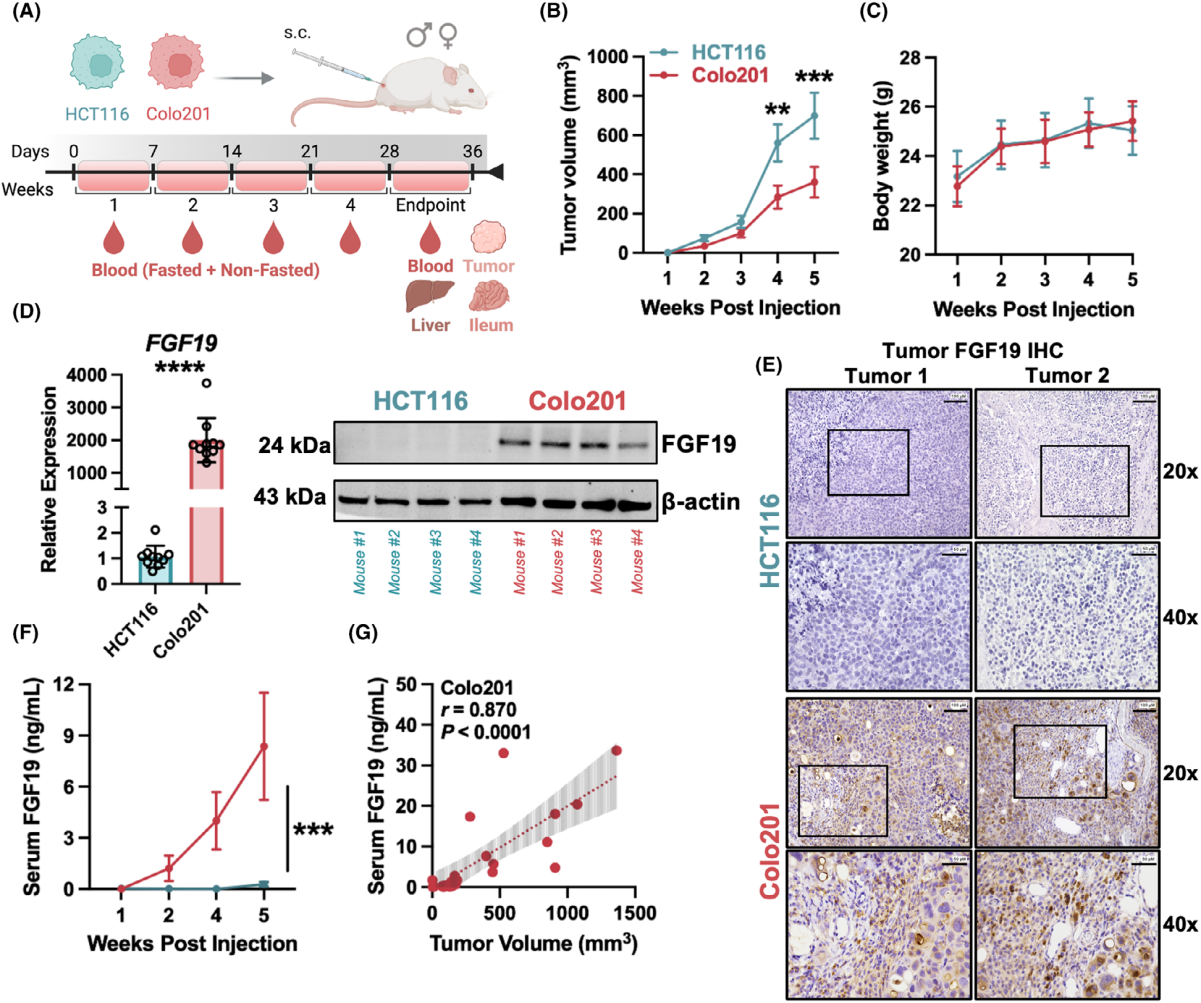
FGF19 is secreted in blood and reflects tumor burden *in vivo*. (A) Schematic detailing the *in vivo* time points and collections in HCT116 and Colo201 CDX models. (B) Caliper measurements of growth for subcutaneous CDXs over time. (C) Mouse weights (G) were not significantly different between CDX groups (*n* = 20–24) throughout study duration. (D) Elevated *FGF19* expression in tumors resected from Colo201 relative to HCT116 CDXs as determined by qRT‐PCR (left) (*n* = 10) and western blot (right). Significance of $2^{-\Delta \Delta C_{T}}$ values was computed via unpaired Student's *t*‐test. (E) Representative immunohistochemical images from resected HCT116 and Colo201 CDXs stained with anti‐FGF19. Images at 20× (top) and 40× (bottom) from two mice of each group (left and right). Scale bars = 100 μm. (F) Quantitation by ELISA of serum FGF19 (ng·mL^−1^) from mice harboring HCT116 (*n* = 9–14) or Colo201 (*n* = 10–14) CDXs measured at weeks after initial injection. (G) Colo201 tumor volume (mm^3^) versus serum FGF19 levels (*n* = 24) at the study endpoint. Simple linear regression line is plotted with 95% confidence intervals and correlation was determined using Spearman analysis. Significance for comparisons between groups of (B, C, F) was computed via a two‐way ANOVA mixed effects model, followed with Šídák's multiple comparisons test. All results are represented as mean ± SEM. S.C., subcutaneous; Statistics: *P < 0.05, **P < 0.01, ***P < 0.001, and ****P < 0.0001.

We next tested the ability of FGF19 to become circulatory if CRC cells were propagated *in vivo*. Because the evaluation of FGF19 in blood circulation has not been well studied regarding CRC progression *in vivo*, we conducted a longitudinal study using a murine subcutaneous CDX model. Mice provide an optimal experimental setting as rodent intestinal cells do not express FGF19, but instead produce the orthologous equivalent Fgf15 [21]. As Fgf15 shares only 51% amino acid sequence identity with its human counterpart, circulating FGF19 can be independently quantitated using specific antibodies [42]. Briefly, equal numbers of age‐matched male and female NSG mice were randomly assigned to two groups to receive subcutaneous injections of HCT116 or Colo201 cells (Fig. 3A). Colo201 cells were selected since they had the highest detectable levels of endogenous FGF19 expression. HCT116 cells were chosen as a FGF19 negative counterpart, and particularly because both Colo201 and HCT116 have reported tumorigenicity in mice [21, 43].

Tumor volume was measurable by week 2 in both CDX groups and progressed throughout the study, with HCT116 cells growing more rapidly than Colo201 (Fig. 3B). Body mass was not significantly different between CDX groups in the weeks following implantation (Fig. 3C). Similar to *in vitro* expression findings in Fig. 2, *FGF19* gene expression was negligible in HCT116 and abundant in Colo201 tumor tissue via qRT‐PCR (*P* = 0.0001) and western blot (Fig. 3D). Protein expression was confirmed by immunohistochemical (IHC) analysis, which showed strong positive staining in Colo201 but not HCT116 tumors (Fig. 3E). Validation of FGF19 antibody specificity for IHC is also shown in human gallbladder tissues, confirming our positive staining (Fig. S3). Furthermore, serum analysis revealed mice with Colo201 CDXs, but not HCT116, had detectable levels of FGF19 in the blood starting 2 weeks post‐injection (Fig. 3F). By week 5, serum FGF19 was significantly higher in Colo201 CDX mice (*P* = 0.0003), which suggests that secreted FGF19 levels are proportional to tumor growth. Endpoint measurements from Colo201 CDX mice comparing serum FGF19 with tumor volumes showed a strong positive correlation (Spearman *r* = 0.870, *P* < 0.0001), thereby suggesting that *in vivo* trends also exhibit cell density‐dependent secretion of FGF19 (Fig. 3G).

### 3.4 Tumor‐derived FGF19 affects liver and diminishes BA metabolism

**Fig. 4:**
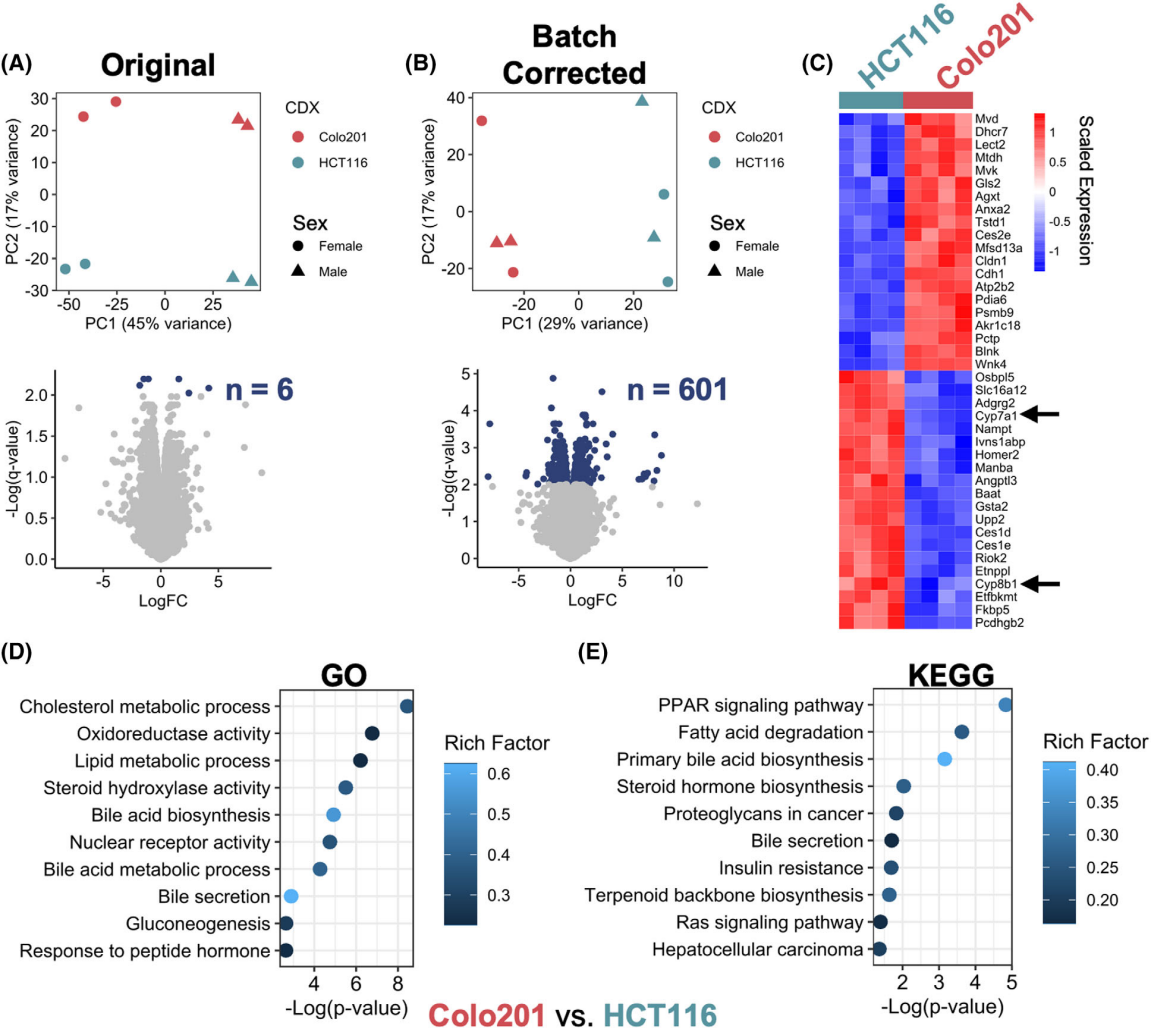
Transcriptomic analysis identifies effects of tumor‐derived FGF19 on murine liver. (A) PCA (top) of original RNA sequencing data demonstrates influence of murine sex (PC1) on transcriptome variability. PC2 variability is explained by the different CDX samples. This large variation results in only 6 genes with an FDR < 1% (navy dots) as demonstrated by volcano plot (bottom). (B) PCA (top) of ComBat‐seq normalized data reveals effective correction of sex‐related batch effects with preservation of PC2 variability. Samples now segregate based on CDX along PC1, resulting in a greater proportion of genes with an FDR < 1% (bottom). (C) Hierarchal clustering of the top 20 up‐ and downregulated DE genes. Black arrows represent FGF19's primary target genes. Samples successfully clustered by CDX group, indicating similarity amongst biological replicates. (D, E) Dot plots illustrating the enrichment of gene ontology (GO) (D) and KEGG terms (E); the top 10 relevant terms were visualized. Rich factor indicates the relative enrichment score, which is calculated based on the number of significant genes compared to expected numbers if genes were randomly distributed.

Since FGF19 acts as a pEH under normal physiological conditions to primarily regulate hepatic *de novo* synthesis of BAs, we tested if circulating FGF19 from aberrant CRC tumor‐derived sources could have systemic effects on the liver. First, RNAseq was performed on murine liver samples obtained from a study of 8 total mice (*n* = 4 per group) using equal numbers of male and female 6–8‐week‐old age‐matched mice that were randomized by cage for receiving subcutaneous HCT116 or Colo201 CDX tumors. Transcriptomic profiles were analyzed using an integrated informatics pipeline. Without batch correction, principal component analysis (PCA) showed ~45% of the observed variance along PC1, which was primarily attributed to sex differences (Fig. 4A). Differences observed between CDX groups explained ~17% of the variance along PC2, resulting in only 6 genes being significant at FDR < 1%. To unmask differences relating to CDX group, sex‐related batch effects were normalized using ComBat‐seq. PCA of batch‐corrected data revealed stabilization of sex‐related heterogeneity with preservation of CDX‐related differences, subsequently unmasking 601 significant genes (Fig. 4B). From this data, hierarchal clustering of the top 20 up‐ and downregulated DEGs confirmed homogeneity amongst biological replicates (Fig. 4C). Importantly, *Cyp7a1* and *Cyp8b1* were noted to be amongst the top DEGs (arrows), showcasing key regulators of BA and cholesterol metabolism as downregulated in the livers from Colo201 compared to HCT116 CDX mice.

Analysis of GO terms (Fig. 4D) and KEGG pathways (Fig. 4E) also revealed an enrichment of GO terms for dysregulated metabolic processes of cholesterols, lipids, BAs, and steroid hydroxylase activity (Fig. 4D) in parallel with PPAR signaling pathway, BA biosynthesis, steroid hormone biosynthesis, and fatty acid degradation KEGG pathways (Fig. 4E). Similar results were obtained using IPA, which predicted these pathways to be interconnected and regulated by NRF2, steroid nuclear receptors (FXR, LXR, and PXR) (Fig. S4A,B), and/or SREBP2‐related binding partners (SCAP and INSIG1) (Fig. S4C), which are all targets of FGF19 signaling [10, 44].

**Fig. 5:**
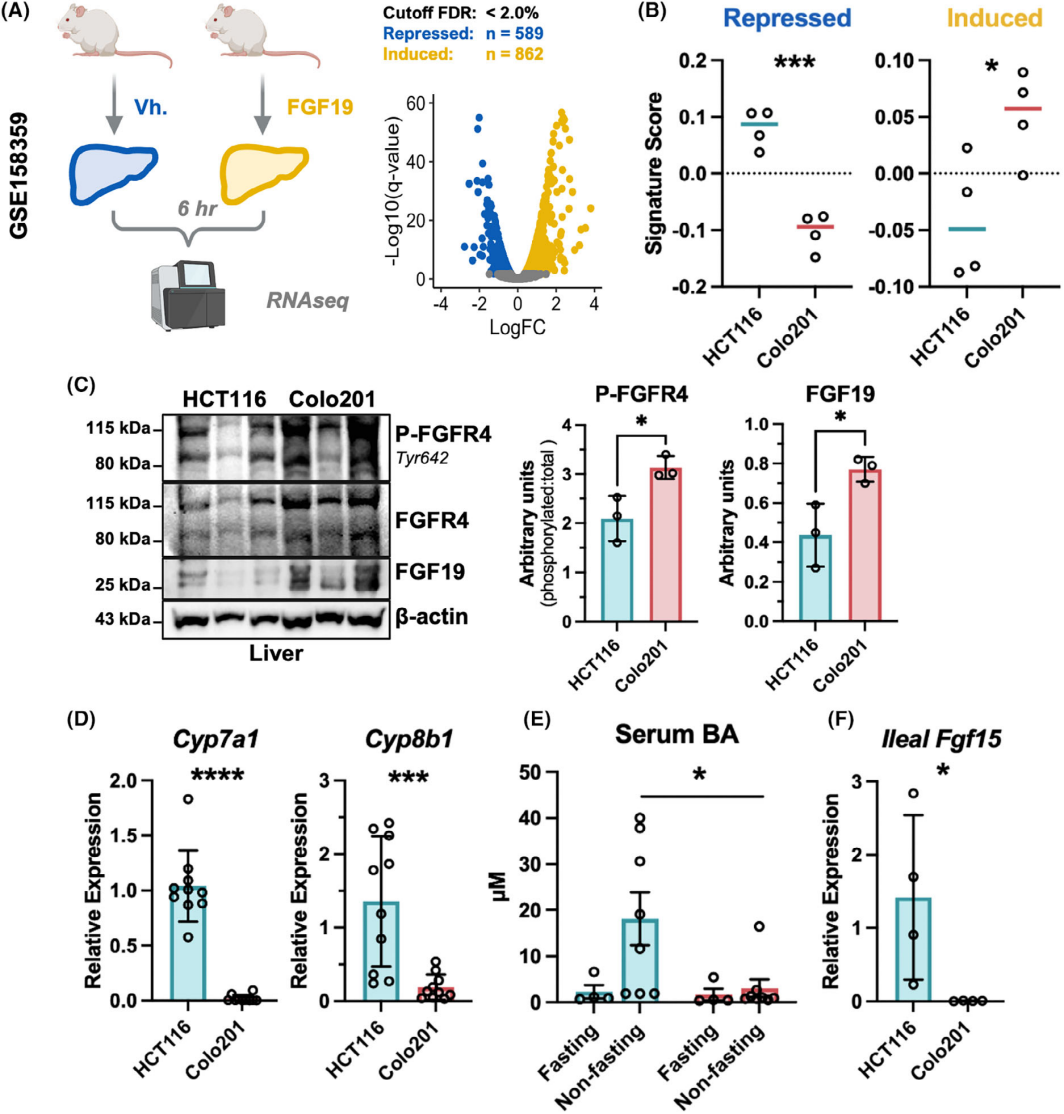
Tumor‐derived FGF19 negatively regulates murine hepatic bile acid synthesis *in vivo*. (A, left) Schematic outlining a prior RNAseq study conducted in GSE158359. Vh indicates vehicle treatment. (A, right) Volcano plot demonstrates the distribution of downregulated (blue) and upregulated (yellow) genes in livers from Vh and FGF19‐treated mice forming the FGF19‐repressed and induced signature sets, respectively. (B) FGF19 signature scores applied to our RNAseq data of livers harvested from HCT116 and Colo201 tumor‐bearing mice. (C) (Left) Western blot of liver tissues from fasted HCT116 and Colo201 CDX mice, *N* = 3. Quantification of western blot proteins, phosphorylated to non‐phosphorylated FGFR4 (right) and total FGF19 (far right). Data points are normalized to respective loading control (β‐Actin) and shown as arbitrary units determined by densitometry. Significance was calculated via unpaired Student's *t*‐test and error bars are ± SD. (D) Quantitative RT‐PCR analysis demonstrates reduced *Cyp7a1* and *Cyp8b1* expression in livers from Colo201 tumor‐bearing mice. (E) Right, total BA levels in serum were low with fasting and upregulated in *ad libitum* fed mice in the HCT116 CDX group (blue); total BA levels in serum were low and remained repressed in *ad libitum* fed mice in the Colo201 CDX group (red). Samples shown were collected from the final time point. Significance for comparisons between CDX group and fasting/fed state were computed via two‐way ANOVA, followed by Tukey's multiple comparisons test. (F) *Fgf15* gene expression was detectable in ileal tissue from HCT116 CDX mice but was repressed in Colo201 CDX mice. (D) and (F) Significance of $2^{-\Delta \Delta C_{T}}$ values were computed via unpaired Student's *t*‐test. Results are represented as mean ± SEM. FDR, False Discovery Rate; Statistics: *P < 0.05, ***P < 0.001, ****P < 0.0001.

To support whether observed molecular changes were primarily associated with FGF19 signaling, we retrospectively compared gene expression trends to a recently published dataset that was generated from RNAseq of livers from FGF19‐treated mice (Fig. 5A) [40]. DE analysis of this dataset revealed 589 downregulated and 862 upregulated DEGs which were deemed unique FGF19‐repressed and induced signatures, respectively. As expected, livers from Colo201 but not HCT116 tumor‐bearing mice were correctly associated with FGF19‐repressed (*P* = 0.0003) and induced (*P* = 0.034) signatures (Fig. 5B).

Results from Colo201‐ and HCT116‐tumor‐bearing mice thus far were suggestive of FGFR4/KLB induced changes in hepatic metabolism, primarily with cholesterol and BA synthesis. To verify tumor‐derived FGF19 is affecting the liver, tissue from fasted mice was used to analyze FGF19 and FGFR4 status via western blot. FGF19 was detected in liver lysates from Colo201 mice and not HCT116, along with increased phosphorylation of FGFR4 which indicates receptor activation (Fig. 5C).

Consistent with our RNAseq data, qRT‐PCR analysis revealed significantly decreased expression of *Cyp7a1* (*P* < 0.0001) and *Cyp8b1* (*P* = 0.042) in livers from mice with Colo201 compared to HCT116 CDX tumors (Fig. 5D). Serum BA levels were also significantly higher (*P* = 0.0378), particularly in non‐fasted (random fed) CDX HCT116 mice compared to Colo201 mice (Fig. 5E). The results showed the undisrupted, normal physiological response of higher serum BA levels in non‐fasted HCT116 CDX mice compared to low serum BA levels in fasted mice. In contrast, mice with the Colo201 tumors did not upregulate serum BA levels under non‐fasted conditions. Mice with HCT116 CDX tumors also exhibited higher expression of ileal *Fgf15* compared to those with Colo201 tumors (*P* = 0.008) (Fig. 5F). Collectively, CDX mice with HCT116 tumors had functional Fgf15‐regulated BA metabolism, but those with high serum FGF19 levels from Colo201 tumors had decreased Fgf15‐regulated BA metabolism.

### 3.5 Tumor‐derived FGF19 affects liver‐to‐body weight ratio

**Fig. 6:**
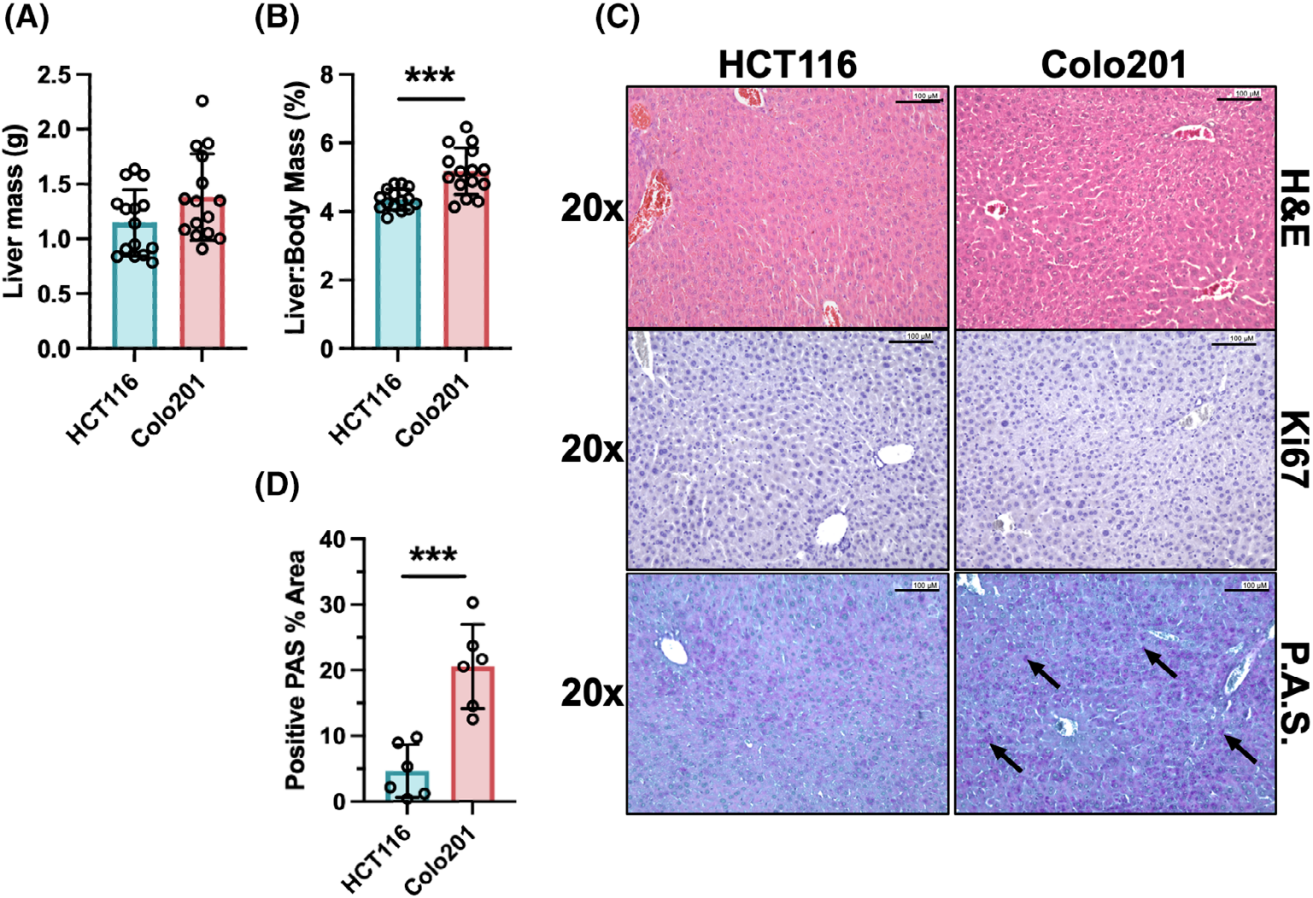
Tumor‐derived FGF19 is associated with an increased liver‐to‐body weight ratio in mice. (A) Mouse liver weights from Colo201 and HCT116 mice were not significantly different. (B) Mouse liver‐to‐body weight ratios were significantly elevated in Colo201 compared with HCT116 CDX mice. (C) Top, hematoxylin and eosin staining of livers. Middle, IHC staining for Ki67 proliferation marker was negative for abnormally proliferating hepatocytes. Bottom, PAS staining showed an increased abundance of purple intracellular granules of glycogen in livers from mice with Colo201 (black arrows) compared to HCT116 CDX. Representative images are at 20x magnification and scale bars = 100 μm. (D) Percent area of positive PAS staining within livers of fasted mice. Quantification was performed on images at 10× magnification from 5 non‐overlapping fields of view per mouse with *n* = 6 mice per group, 3 males and 3 females. Unpaired Student's *t*‐test was used to compute significance for all analyses. Results reported as mean ± SD. Statistics: ***P < 0.001.

Direct liver weight comparisons showed no significant differences between CDX groups (Fig. 6A), except when liver‐to‐body weight percent ratios were calculated, revealing that Colo201 CDXs levels were significantly increased compared to HCT116's (difference between means 0.8244 ± 0.1927, *P* = 0.017) (Fig. 6B). The increased liver‐to‐body weight ratio is likely not due to enhanced hepatocyte proliferation, because IHC of marker of proliferation, Ki67, did not show positive staining amongst liver tissues, nor did it show increased expression levels (Fig. 6C, Figs S3 and S5B). However, FGF19's role in regulating hepatic glycogen pathway activity has been shown to contribute to increased hepatic mass in the absence of abnormal hepatocyte proliferation [45]. Periodic Acid‐Schiff (PAS) staining of the livers was performed to indicate the glycogen as granules within the parenchyma. Staining of fasted male and female livers showed greater glycogen presence in Colo201 CDXs compared to HCT116 as determined by quantification of percent area positive PAS staining (mean = 20.57 ± 7.39; mean 4.643 ± 5.108, respectively, *P* = 0.0004) (Fig. 6C). In further support for tumor‐derived FGF19 induction of metabolic perturbations, immunoblots were used to assess the expression of glycogen synthase kinase, GSK‐3α/β, which in its non‐phosphorylated form maintains inhibition of glycogen synthesis via phosphorylation of glycogen synthase. Upon GSK‐3α/β's phosphorylation in response to nutrient sensing, its inhibition of glycogen synthase is relieved, and glycogen synthesis from glucose is permitted. Results showed livers from mice with Colo201CDX exhibited similar levels of phosphorylated GSK‐3α/β in fasting and non‐fasting states (mean difference = 0.0333, *P* = 0.7666). In contrast, livers from mice with HCT116 CDX had significantly greater GSK‐3α/β phosphorylation with non‐fasted compared to fasted states (mean difference = 0.2653, *P* = 0.0403) (Fig. S5A). The atypical activity of glycogen synthesis during fasting, increased glycogen stores within hepatocytes, unaltered expression of proliferation markers, and enhanced liver‐to‐body weight ratio, collectively show that tumor‐derived FGF19 alters metabolic‐associated molecular pathways within the liver.

## 4 Discussion

Blood‐based approaches for screening, diagnosing, and monitoring CRC have been a long‐standing goal to reduce disease morbidity and mortality. Herein, we identified FGF19 to be a plausible serum marker for CRC that is secreted by tumors that have elevated FGF19 expression and that exerts endocrine effects on liver tissue.

FGF19 is a non‐canonical member (along with FGF21 and FGF23) of the FGF family. FGF19 production is restricted temporally during the postprandial state and spatially to ileocytes and gallbladder epithelial cells in healthy individuals [46]. Despite not being produced in colon, FGF19 overexpression was a common feature of polyps and CRC, as well as other cancers including lung, liver, and prostate [7, 9, 13, 15, 47]. Collectively, these properties contribute to the attractiveness of FGF19 as a candidate marker for CRC. In addition, we found through a meta‐dataset analysis that overexpression of FGF19 was associated with unfavorable clinical prognosticators such as disease progression, treatment failure, and poor survival. Our meta‐dataset and TCGA‐COAD also shared negative correlations between *FGF19* expression and immune and stromal scores determined by ESTIMATE analysis. Statistical significance and modest rho values (≤ 0.2) suggest similar correlations in those datasets, in that FGF19 is inversely associated with immune infiltration/activity and stromal/fibrotic TME features in both datasets. The ESTIMATE analysis also showed that the association was more specific to the immune score relative to stromal score in specifically the carcinoma subset from the meta‐dataset. These results are unsurprising given previous studies delineating FGF19's role in promoting colorectal tumorigenesis, epithelial to mesenchymal transition (EMT), and chemoresistance through pro‐oncogenic interactions with FGFR4 [28]. Therefore, it is possible that detection of CRC‐derived FGF19 in patient serum could be an important indicator of prognosis and/or tumor chemosensitivity to recently developed FGFR4 kinase inhibitors Roblitinib and Fisogatinib [48, 49].

Although FGF19's expression profile in CRC supports its strength as a potential biomarker, translatability to its detection in blood serum has remained untested. To address this, we first tested a panel of CRC cell lines for FGF19 expression and secretion. FGF19 was expressed and secreted by CRC cells at levels coinciding with cell density. FGF19 secretion patterns according to cell density were readily recapitulated *in vivo* using subcutaneous xenografts of FGF19‐weak (HCT116) and FGF19‐strong (Colo201) CRC cell lines. In the animal models, we observed greater tumor volumes in HCT116 xenografts compared to Colo201, which is consistent with their reported doubling rates in culture for HCT116 being 25 h versus 41 for Colo201 [50].

FGF19 was detected in the serum of Colo201 tumor‐bearing mice at levels that were dependent on tumor volume and immunohistochemical staining showed that Colo201 CDX tumors were substantially positive for FGF19 expression. Collectively, these findings indicate that detection of FGF19 in the blood was dependent on the relative expression in the CRC tumor cells, and for CRC cells that had FGF19 expression, blood levels were at least in part associated with the tumor volume. Finally, we noted that serum FGF19 levels from Colo201 had concentrations more than 10 ng·mL^−1^, which is substantially greater than typical peak postprandial levels in humans (up to ~0.6 ng·mL^−1^) [3]. Such findings, when combined with the observation that CRC cells produce FGF19 in the absence of stimulatory factors, may point to elevated serum FGF19 potentially being found in fasted CRC patients where levels are normally unquantifiable [51].

Based on the detection of supraphysiologic serum FGF19 levels in mice, we speculated whether tumor‐derived FGF19 exerted endocrine effects on the liver, its primary target organ. This is important as clinical manifestations, collectively termed ectopic hormone syndromes, could further facilitate CRC diagnosis and management as is the case for other neoplastic diseases like Zollinger‐Ellison syndrome and Cushing's disease [52]. Therefore, we evaluated global molecular changes in livers obtained from mice harboring HCT116 or Colo201 CDXs via RNAseq. In line with FGF19's known physiologic role regulating *de novo* BA synthesis, corresponding genes and pathways involved in cholesterol and BA metabolism were strongly modulated in livers from mice with Colo201 versus HCT116 CDXs. This was evidenced by decreased expression of genes for steroid hydroxylase enzymes *Cyp7a1* and *Cyp8b1* as well as enrichment of related GO terms (metabolic processes for cholesterol, lipid, Bas, and oxidoreductase, steroid hydroxylase, and nuclear receptor activity) and KEGG pathways (PPAR signaling, primary BA and steroid hormone biosynthesis, BA secretion, insulin resistance, and terpenoid backbone biosynthesis). The most significantly enriched canonical pathway predicted by IPA was Nrf2‐mediated oxidative stress response, which has been shown to be a mechanism by which FGF19 enables tumor cell survival in HCC [10]. Interestingly, the subsequent enriched pathways predicted by IPA to be significantly activated were four pathways regarding cholesterol biosynthesis and a pathway for FXR/RXR activation. Corroborating these five IPA pathway predictions, heatmap DEGs *Mvk, Mvd*, and *Dhcr7* were significantly upregulated (encodes for key enzymes in cholesterol biosynthesis), and *Cyp7a1*, *Cyp8b1*, and *Baat* were significantly downregulated (encodes for enzymes in BA synthesis and BA conjugation) which would be downstream targets for FXR/RXR. In addition to the metabolic alterations, other KEGG‐enriched pathways were proteoglycans in cancer, Ras signaling pathway, and hepatocellular carcinoma.

A novel finding from our study was the deregulation of total BA levels in serum from male mice with elevated tumor‐derived FGF19. Total serum BAs in *ad libitum* fed mice were repressed to levels on par with fasted mice with high‐FGF19. Compared to males, female mice have a larger total BA pool, greater conversion of cholesterol to BA, and demonstrate less remarkable changes in cholesterol levels during typical fasting versus fed conditions [53, 54, 55, 56]. Therefore, to examine the physiological impact of FGF induced repression on BA production from cholesterol when serum levels are anticipated to be low versus high, females were not optimal models for comparison. Overall findings were that total serum BAs in *ad libitum* fed male mice were repressed to levels on par with fasted mice in high‐FGF19 Colo201 tumor group, whereas serum BA levels in low‐FGF19 HCT116 tumor‐bearing mice were significantly higher with random‐feeding compared to fasting. These data provide supporting evidence that tumor‐derived FGF19 can repress normal physiological levels of total BAs in mouse serum. Moreover, we showed low ileal Fgf15 expression in Colo201 mice with high‐FGF19, therefore providing a logical link for the disruption of normal physiological enteroendocrine signaling from the ileum to the liver by tumor‐derived FGF19.

The BA assay that was used in our study did not specify which BAs were affected. Previous work has shown that mice administered human FGF19 shifted their primary BA synthesis pathway from classic synthesis of cholic acid (CA) to the alternative pathway that directly produces chenodeoxycholic acid (CDCA) and muricholic acid (MCA) [57, 58]. In mice, muricholic acid (MCA) predominates the murine BA pool, and its secondary BAs are less toxic and hydrophobic than those from CDCA. In humans, the secondary BAs produced from CDCA are deoxycholic acid (DCA) and lithocholic acid (LCA), which have been shown to be cytotoxic and cancer promoting [57]. As pointed out in this review paper, comparisons between specific BAs in humans and mice are difficult due to these species‐specific discrepancies and are incongruent in the context of mice engrafted with human CRC tumors expressing FGF19 [53].

FGF19's effects on liver‐to‐body weight ratios have been attributed to increased protein synthesis, glycogen accumulation, and tumor formation, and the mouse Fgf15 counterpart has been associated with liver weight during tissue regeneration post‐resection [59]. Mice injected with a single dose of FGF19 had elevated liver‐to‐body weight ratio and exhibited increased expression of *Ki67*, *Afp*, and *Gpc3* after 24 weeks in livers of diabetic *db/db* mice, and after 12 months in mice with diet induced obesity [60, 61]. Transgenic mice with ectopic FGF19 expression in skeletal muscle showed histological features of dysplastic foci at 7–9 months and developed HCC tumors in ~53% of mice by 10–12 months [8]. This current study was conducted over a 5‐week time frame and the findings of significantly increased liver‐to‐body weight ratios in mice with Colo201 tumors provided evidence that ectopic tumor‐derived FGF19 also results in pathological effects in the liver. With this shorter study duration, induction of neoplastic lesions or hepatocyte proliferation within the livers was not detected, although tumor‐derived FGF19 effects were consistent with aberrant hepatic metabolism, most notably on glycogen synthesis and accumulation. A prior study found that FGF19 enhances production of glycogen in the liver through Ras–ERK‐p90RSK downstream of FGFR4 and has also been shown to decrease gluconeogenesis via insulin‐independent mechanisms [45]. The livers of Colo201 mice exhibited increased glycogen staining and fasted mice showed phosphorylated GSK‐3α/β in liver during fasting, which would permit glycogen synthesis even in the absence of glucose intake.

A limitation of our study is that different CRC cell lines were used in our CDX models to test whether tumor‐derived FGF19 is detectable in blood, rather than using the same cell line with genetic manipulation of FGF19. To our knowledge, Colo201 has not been published in any studies with successful knockdown or knockout of FGF19, suggesting that it is crucial for survival in these cells. Our attempts to knockdown FGF19 through lentivirus delivery of shRNA resulted in slowed cell proliferation and lacked viable clonal expansion when cells with high endogenous FGF19 expression were subjected to the knockdown FGF19 strategy. We have successfully overexpressed FGF19 in HCT116 cells and confirmed increased FGF19 expression *in vitro* but have not been successful in performing *in vivo* studies in part due to premature ulceration of the subcutaneous tumors. Since our purpose was to evaluate detection of FGF19 *in vivo* but not to functionally manipulate FGF19, we selected Colo201 and HCT116 from our CRC cell line panel based upon their divergent FGF19 expression and their reported *in vivo* tumor formation. Interestingly, the cell lines were the subject of predecessor publications that used HCT116 and Colo201 to study FGF19/FGFR4 [21, 24]. The current study differs in that we measured serum FGF19 in both male and female mice and saw significant correlation of FGF19 levels with Colo201 tumor growth that expressed endogenously high levels of FGF19 but not HCT116 with low FGF19 expression, suggesting a clinical utility of FGF19 for blood‐based tumor monitoring. The current study also has novelty from previous studies by demonstrating FGF19 is a pEH secreted by human tumors that can activate murine hepatic FGFR4 and modulate downstream metabolic pathology. Immune deficient mice were required to allow for human CDX engraftment, so a limitation is that the tumor microenvironment cannot be engineered to precisely recapitulate the bioinformatic predictions for CRC patients. These limitations highlight the need for continued exploration of FGF19 as a CRC serum marker.

## 5 Conclusions

Collectively, the importance of this study is centered on the detection of secreted FGF19 in the blood serum from CRC tumors with high FGF19 expression, and alterations of the molecular landscape by tumor‐derived FGF19 to disrupt normal hepatic physiology. The findings indicate that symptoms may manifest clinically as it relates to suppressed BA recirculation, such as chronic diarrhea or weight loss stemming from dietary lipid malabsorption [62, 63]. These are both associated with CRC and thus could be indicators of elevated FGF19 levels in cancer patients. Results further show that tumor‐derived FGF19 actively modifies hepatic physiology at the molecular level in an endocrine manner, and this study overall highlights serum FGF19 as being worthy of further investigation to delineate translational utility.

## Conflict of interest

The authors declare no conflict of interest.

## Author contributions

J.M.B. and M.W.R. conceptualized the research, performed experiments, and wrote the manuscript. J.A.G. assisted with all experiments and critically evaluated the manuscript. J.M.B. and S.P.N. performed immunohistochemical analyses and critically evaluated the manuscript. J.G.P. provided pathology expertise for histology. D.A.A. conceptualized the research, contributed to the writing, and critically evaluated the manuscript.

## Supporting information


**Fig. S1.** Study outline and associated informatics pipeline for *in vivo* RNAseq data.

**Fig. S2.** ESTIMATE scores are inversely correlated with *FGF19* expression in CRC.

**Fig. S3.** Distribution of FGF19 and Ki67 immunohistochemistry staining in tissue controls.

**Fig. S4.** Prediction of pathways networks using ingenuity pathway analysis.

**Fig. S5.** Increased liver‐to‐body weight ratio is likely due to abnormal glycogen production instead of hepatocellular proliferation.

## Data Availability

The datasets generated for this study can be found in the Array Express (E‐MTAB‐10089) and the Gene Expression Omnibus (GSE188745).
