# Supplementary figures and images for: Colorectal cancer‐derived FGF19 is a metabolically active serum biomarker that exerts enteroendocrine effects on mouse liver
Source: Mol Oncol. 2026 Feb 3;20(6):1494–512. doi: 10.1002/1878-0261.70212 (PMC13238793; doi:10.1002/1878-0261.70212)

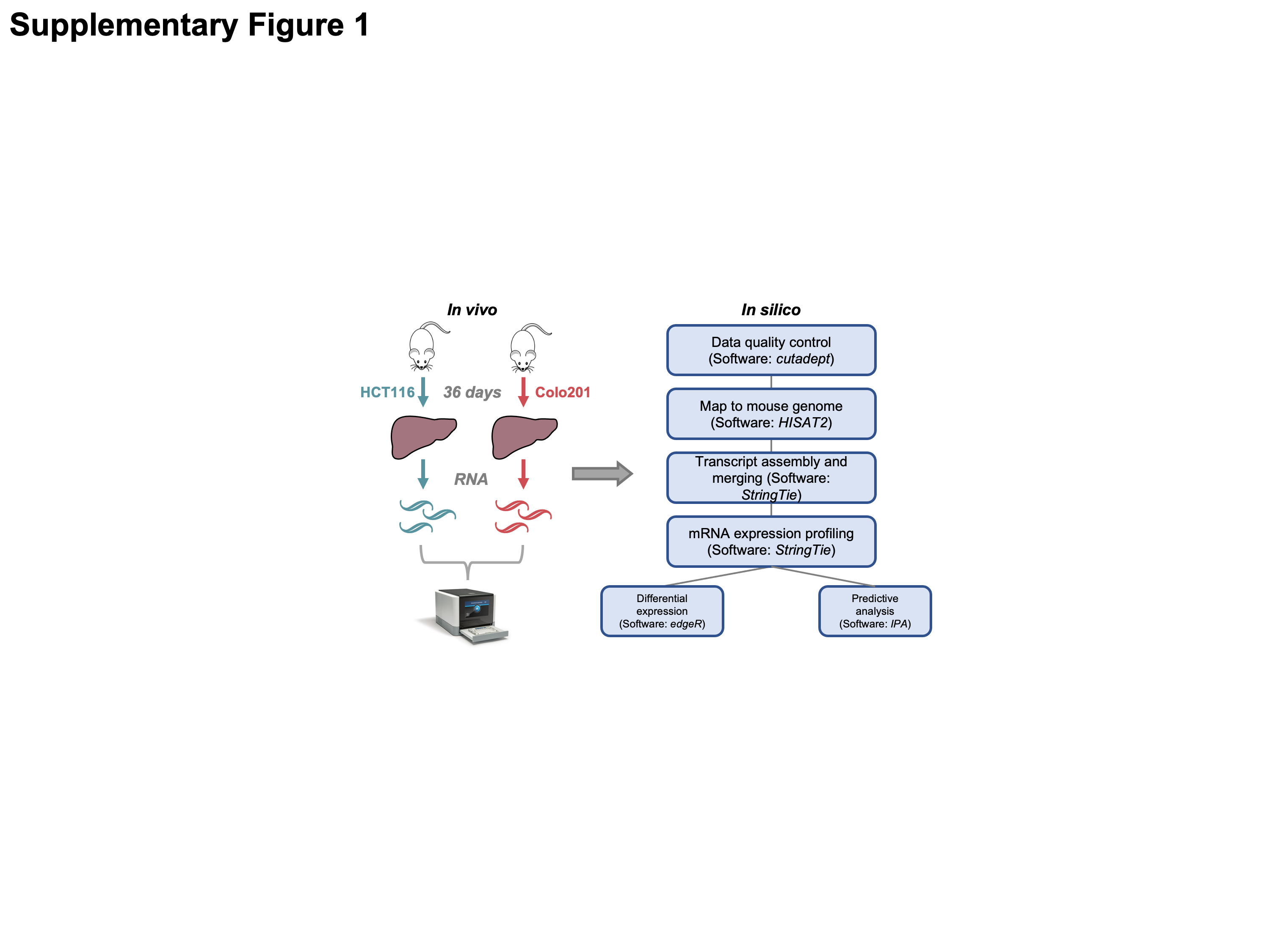

Supplement: Supplementary file 1 — Fig. S1. Study outline and associated informatics pipeline for in vivo RNAseq data. [file MOL2-20-1494-s004.tiff]

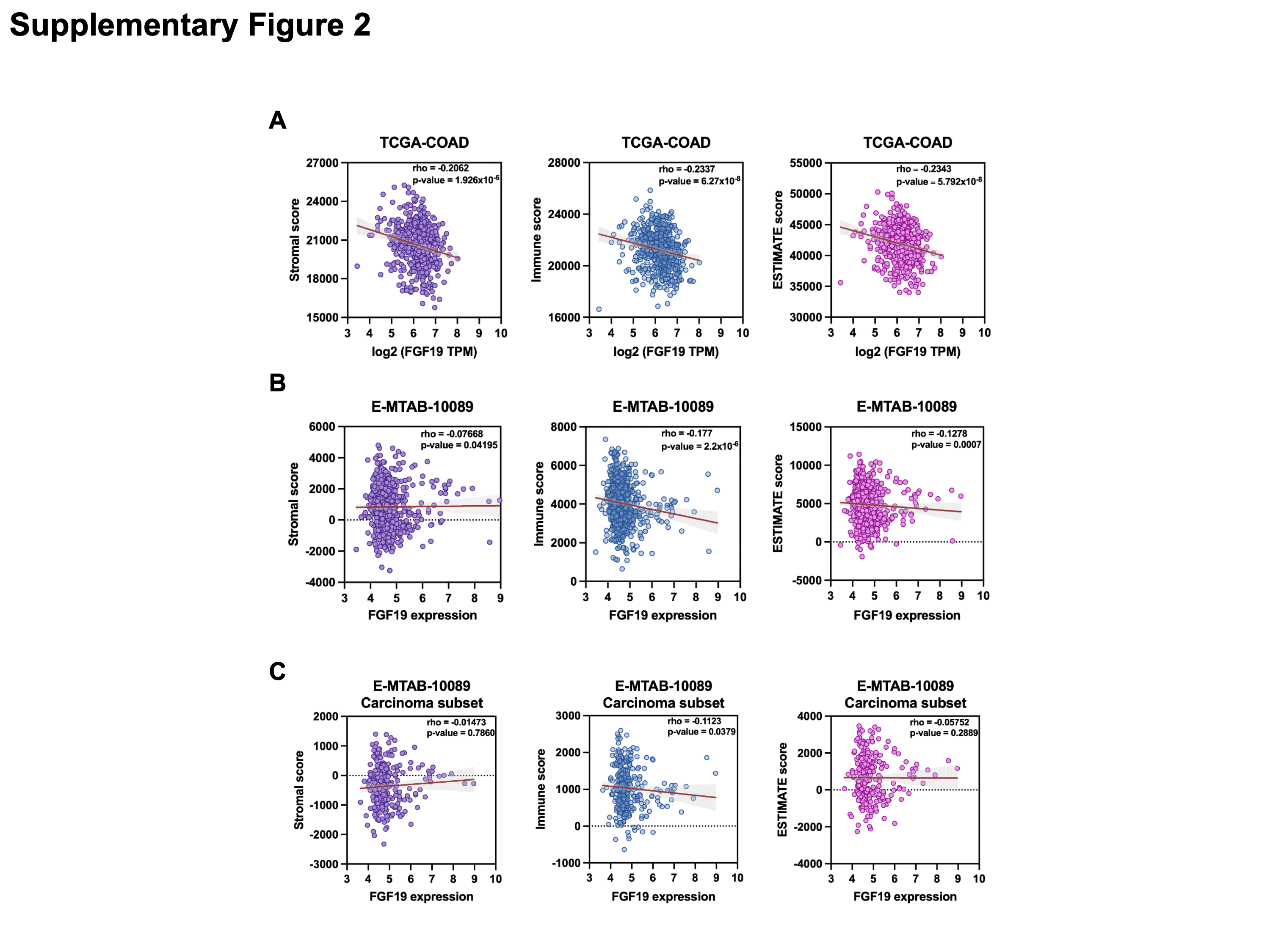

Supplement: Supplementary file 2 — Fig. S2. ESTIMATE scores are inversely correlated with FGF19 expression in CRC. [file MOL2-20-1494-s003.tiff]

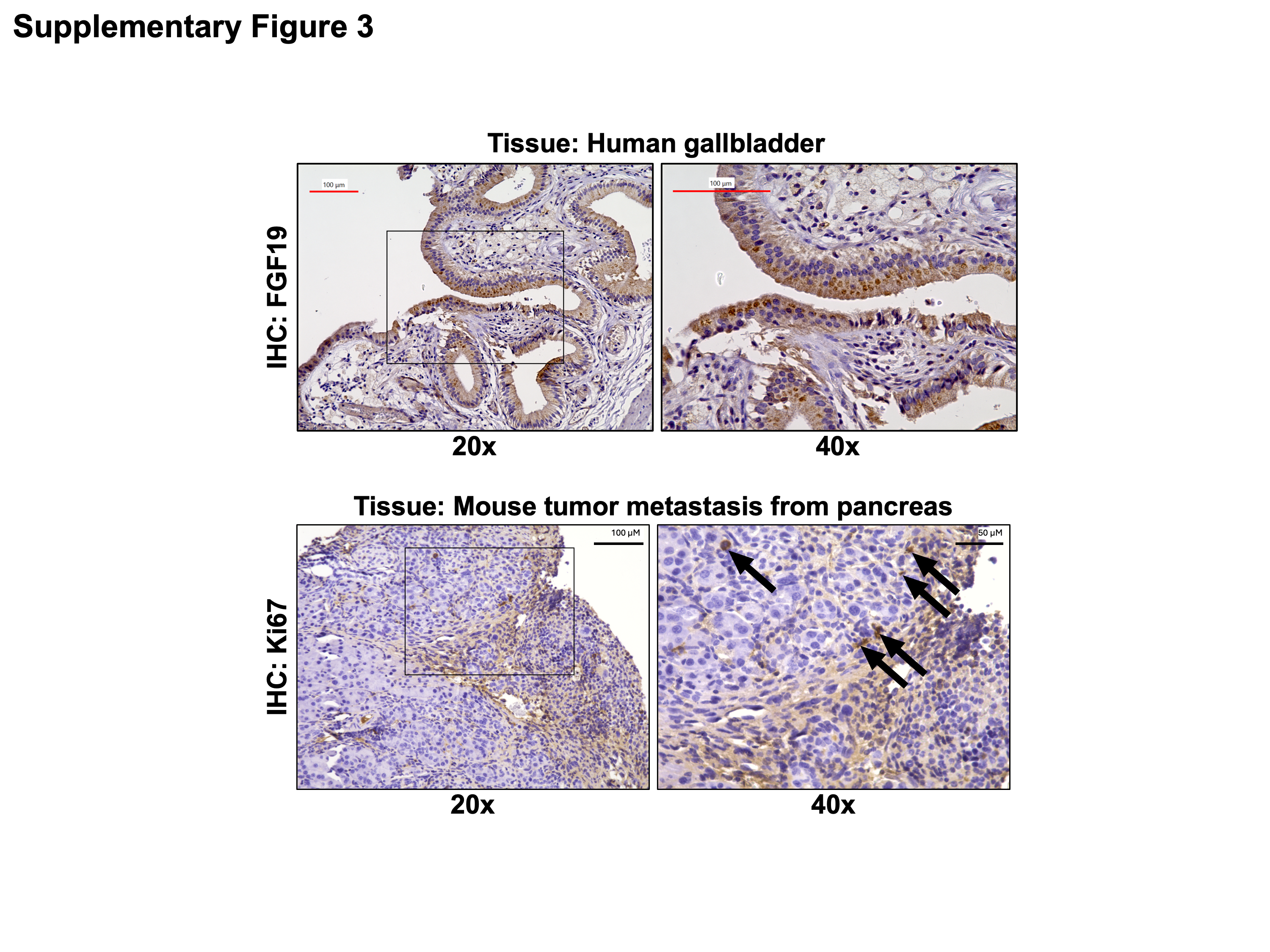

Supplement: Supplementary file 3 — Fig. S3. Distribution of FGF19 and Ki67 immunohistochemistry staining in tissue controls. [file MOL2-20-1494-s002.tiff]

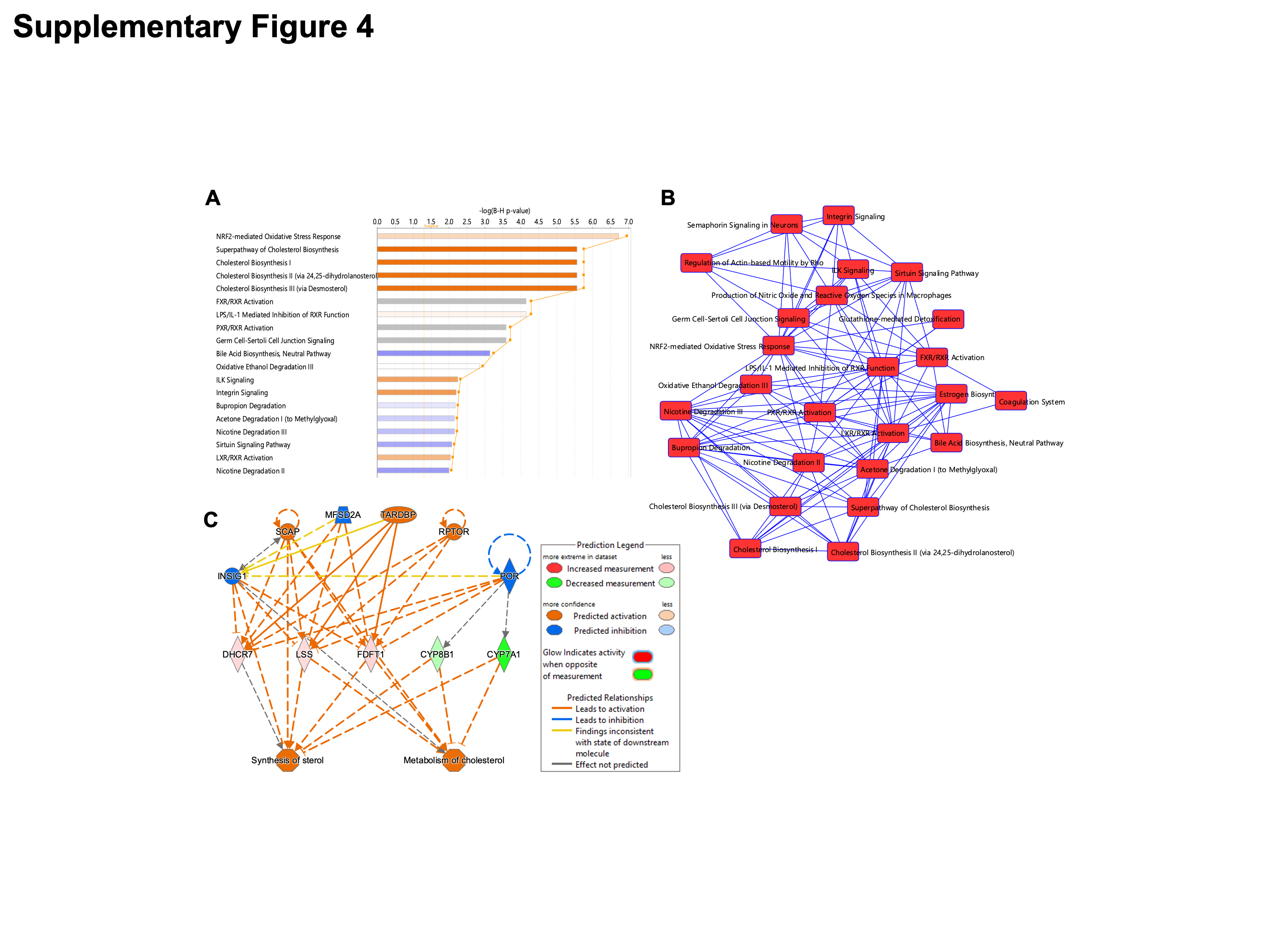

Supplement: Supplementary file 4 — Fig. S4. Prediction of pathways networks using ingenuity pathway analysis. [file MOL2-20-1494-s001.tiff]

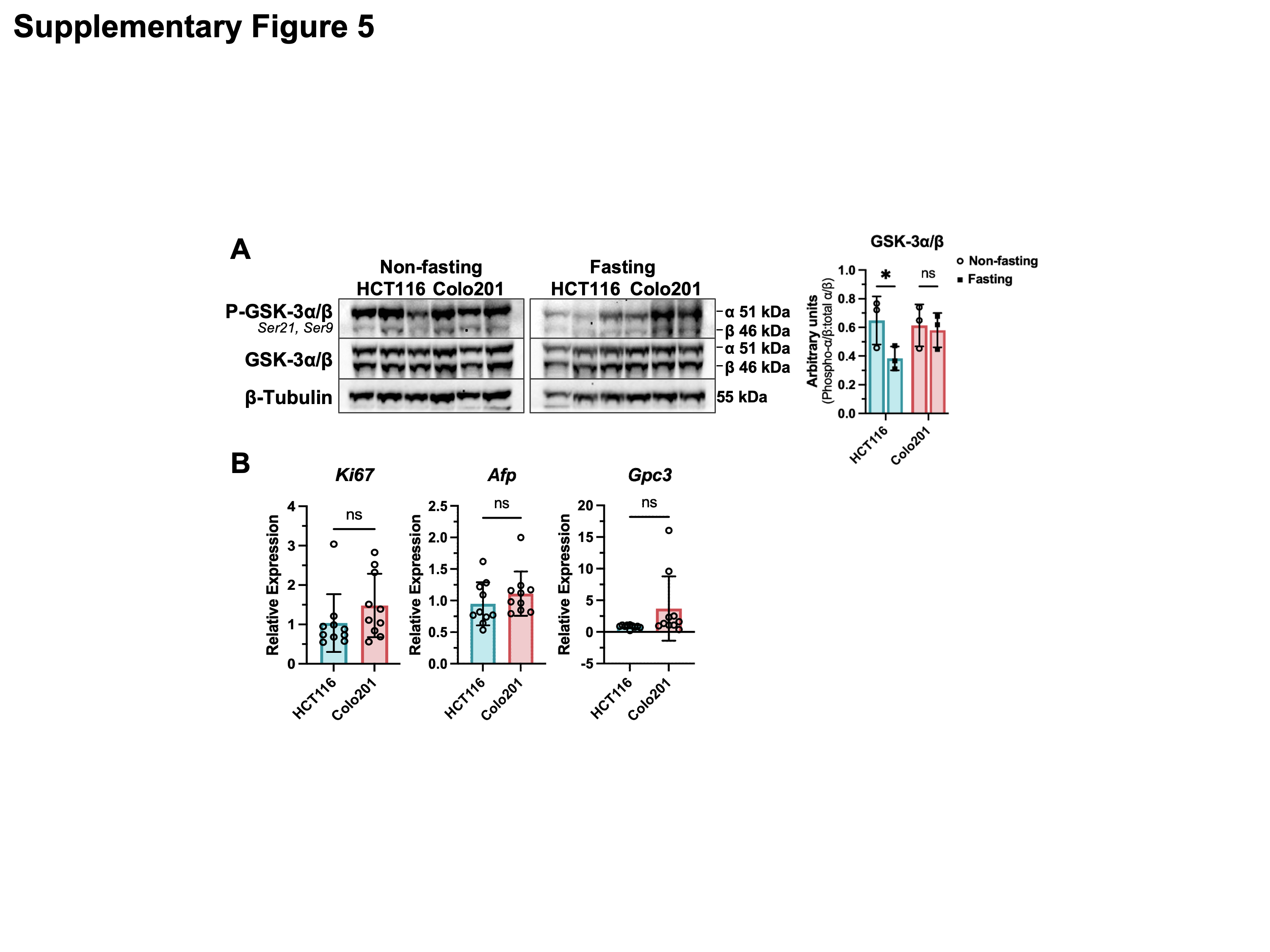

Supplement: Supplementary file 5 — Fig. S5. Increased liver‐to‐body weight ratio is likely due to abnormal glycogen production instead of hepatocellular proliferation. [file MOL2-20-1494-s005.tiff]
